# Association between the planetary health diet and sleep health in older adults: findings from a national community-based study

**DOI:** 10.3389/fnut.2026.1758298

**Published:** 2026-02-04

**Authors:** Xinyan Ma, Yuhong Zhao, Hanqing Zhao, Daan Zhou, Hao Ai, Minghui Sun

**Affiliations:** 1 The Third Affiliated Hospital of Jinzhou Medical University, Jinzhou, China; 2 Department of Clinical Epidemiology, Shengjing Hospital of China Medical University, Shenyang, China; 3 Liaoning Key Laboratory of Precision Medical Research on Major Chronic Disease, Shenyang, China; 4 Hunan Provincial Center for Disease Control and Prevention, Changsha, China; 5 Liaoning Provincial Key Laboratory of Follicular Development and Reproductive Health, Jinzhou, China

**Keywords:** diet, older adults, planetary health diet, sleep duration, sleep quality

## Abstract

**Background:**

The planetary health diet (PHD) has been established as linked to various diseases, yet its association with sleep health remains unexplored. This study is the first to investigate the associations of the PHD with sleep quality and duration, and to further evaluate its joint association with physical exercise, leveraging a national sample to inform healthy aging strategies.

**Methods:**

This study utilized data from 9,041 elderly participants in the eighth wave of the Chinese longitudinal healthy longevity survey. A PHD Index (PHDI) was constructed based on 10 food groups from a simplified food frequency questionnaire, while sleep quality and duration data were obtained through face-to-face interviews. Multivariable logistic regression models were employed to examine the associations between the PHD and sleep quality and duration.

**Results:**

The study identified 4,730 (52.32%) participants with good sleep quality and 3,257 (36.02%) with adequate sleep duration. In the adjusted model, higher PHDI scores were associated with better sleep quality (OR = 1.35; 95% CI = 1.20–1.52) and adequate sleep duration (OR = 1.36; 95% CI = 1.20–1.54). Each one-point increase in the PHDI score was associated with 5 and 4% higher odds of good sleep quality and adequate sleep duration, respectively. Notably, in the joint analysis, participants with both a high PHDI and regular physical exercise exhibited significantly higher odds of good sleep quality (OR = 1.64; 95% CI: 1.42–1.89) and adequate sleep duration (OR = 1.48; 95% CI: 1.27–1.73) compared to those with a low PHDI and no exercise.

**Conclusion:**

Our findings indicate that a higher PHD was associated with better sleep quality and adequate sleep duration, highlighting the dual benefits of plant-based and sustainable diets for personal sleep health and the planet.

## Introduction

Compelling evidence links poor sleep health to an increased risk of various chronic conditions, encompassing cardiovascular and cerebrovascular diseases, mental disorders, and cognitive impairment (1). The implications of this relationship were particularly profound for older adults, who experience a greater burden from these sequelae (2). Epidemiological studies indicate that more than one-third of the U.S. population experiences sleep-related health problems (3). In China, sleep health issues affect up to 70% of older adults (4). These conditions significantly compromise physical health, elevating risks of chronic diseases and mortality (5). In light of this, addressing sleep health is critical for developing public health strategies to promote healthy aging and reduce mortality. As part of this effort, focus is turning to diet as a key modifiable factor for disease prevention.

The Planetary Health Diet (PHD) was proposed by the EAT-Lancet Commission in 2019 to promote optimal human nutrition and global planetary health (6). This dietary pattern emphasizes the consumption of plant-based foods, including fresh vegetables, fruits, nuts, and legumes, whereas it recommends limited intake of red meat, dairy products, and added sugars (7). While previous research has indicated links between the PHD and various diseases, such as cardiovascular disease (8), cancer (9), and metabolic disorders (10), its relationship with sleep health remains unclear. However, emerging evidence suggests that the PHD may have a profound impact on sleep health, which was a key pillar of overall health. The potential mechanisms may involve the regulation of circadian rhythms through the provision of micronutrients (e.g., magnesium, tryptophan), anti-inflammatory properties of dietary components, and gut microbiota regulation by dietary fiber (11–13).

Beyond dietary factors, physical exercise was also recognized as a beneficial non-pharmacological intervention for improving sleep health among older adults (14). However, evidence regarding the joint association of adherence to the PHD and physical exercise with sleep outcomes remains scarce.

Therefore, this study aims to investigate the associations of PHD adherence with sleep quality and duration in the Chinese Longitudinal Healthy Longevity Survey (CLHLS) cohort, and to evaluate the joint associations of PHD and physical exercise on sleep health.

## Methods

### Study design and participants

The CLHLS was a nationwide, population-based prospective study that systematically investigates determinants of health in Chinese adults aged ≥65 years. It collects comprehensive data on demographics, lifestyle (e.g., diet, social engagement, sleep, physical activity), mental status, and physical measurements through face-to-face interviews and examinations conducted by trained staff. For this analysis, we used data from the eighth wave of the CLHLS. A detailed description of the CLHLS methodology has been published previously (15, 16).

**Figure 1:**
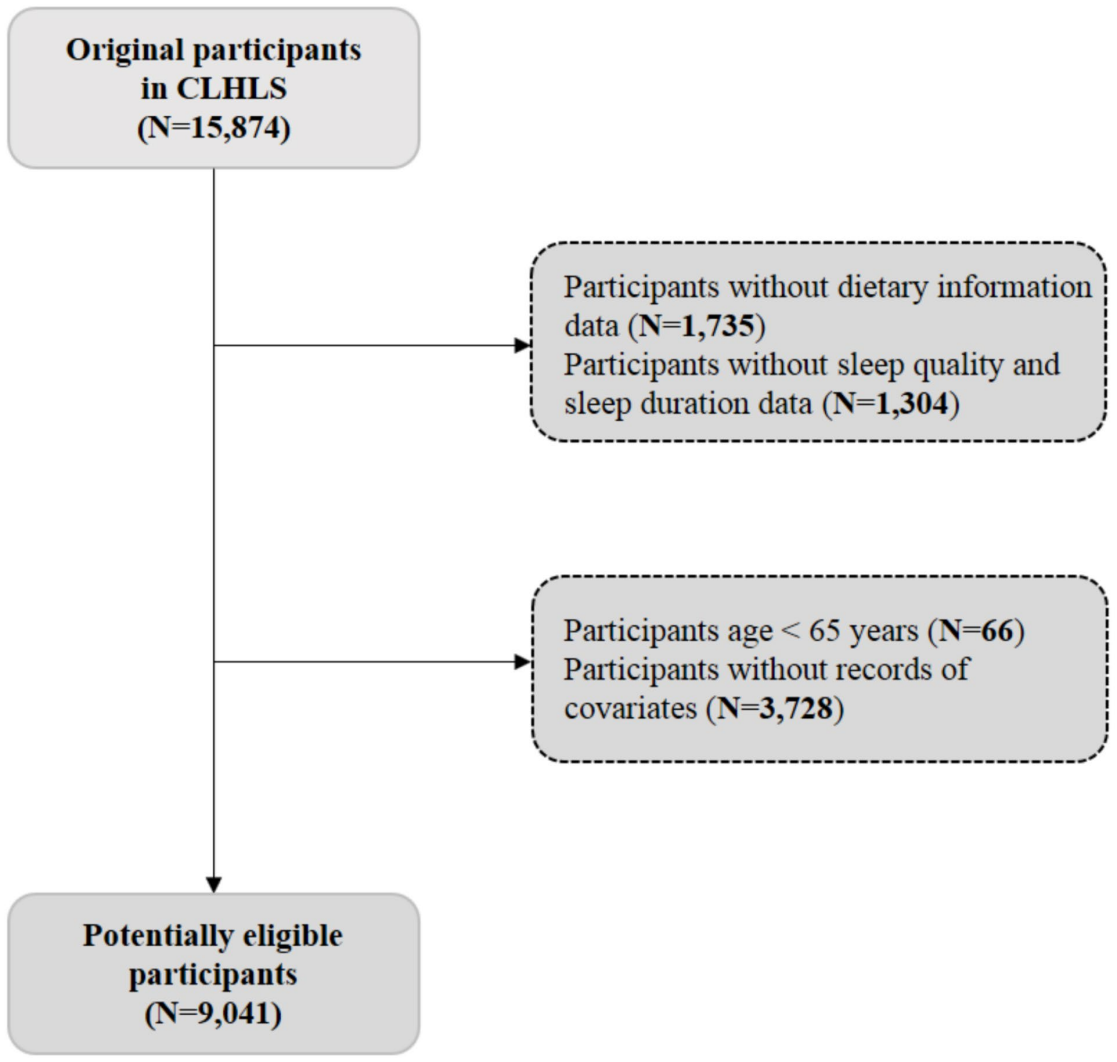
Flow chart of the study participant selection process.

A total of 15,874 participants were initially enrolled. Exclusions were made for individuals with: (1) incomplete dietary intake information; (2) incomplete sleep health data; (3) a baseline age of less than 65 years; and (4) missing covariate data. Finally, the analytic sample for this study comprised 9,041 older adults (Figure 1). The protocol for the CLHLS was approved by the Biomedical Ethics Committee of Peking University (IRB00001052-13074).

### Dietary assessment

Dietary intake was assessed using a simplified food frequency questionnaire (FFQ), a validated instrument widely employed in prior studies (17, 18). It collected data on the consumption of key food groups, including fresh fruits, vegetables, fish, meat, eggs, soy products, dairy, and sugars etc. The frequency of consumption was categorized differently for specific foods: for instance, fruits and vegetables used the options “almost daily,” “often,” “occasionally,” or “seldom/never.” For other food groups, the categories were “almost daily,” “not daily but at least weekly,” “not weekly but at least monthly,” “not monthly but occasionally,” and “seldom/never.” Data on staple food types and quantities were also recorded.

Adherence to the PHD was assessed using the PHD Index (PHDI), which was constructed from 10 food components in the simplified FFQ, in accordance with established methodologies (19, 20). The PHDI includes food groups categorized into two types: those encouraged for higher consumption (e.g., fresh vegetables, fruits, legumes, nuts, whole grains, and fish) and those recommended in moderation (e.g., eggs, meat, dairy, and sugars). Each food group was assigned a score ranging from 0 to 3 based on consumption frequency, with detailed scoring criteria provided in Supplementary Table S1.

### Assessment of sleep health

In this study, sleep health was assessed using measures of sleep quality and duration. Sleep quality, assessed by the question “How do you rate your current sleep quality?” Responses were collapsed into two categories: “good” (“very good” or “good”) and “poor” (“fair,” “poor,” or “very poor”) (21). Sleep duration, assessed by “How many hours of sleep do you get per day?”, was first categorized as short (<7 h), optimal (7–8 h), or long (>8 h) per National Sleep Foundation guidelines (22). For the purposes of statistical modeling, sleep duration was dichotomized into adequate (7–8 h) versus inadequate (<7 or >8 h).

### Assessment of covariates

Our analysis adjusted for a wide range of potential confounders spanning demographic, socioeconomic, lifestyle, and health factors. These included age, gender (men/women), ethnicity (Han/other), residence area (city/town/rural), annual income (≥30,000/<30,000 yuan), marital status (cohabiting with a partner or not), current labor status, exercise, alcohol consumption, smoking status (all yes/no), body mass index (BMI), hypertension, and diabetes. BMI was calculated as weight in kilograms divided by the square of height in meters. Hypertension was defined as an average systolic/diastolic blood pressure ≥140/90 mmHg from two consecutive measurements and/or self-reported physician diagnosis; whereas diabetes mellitus was determined solely by self-reported diagnosis.

### Statistical analyses

The normality of continuous variables was assessed with the Kolmogorov–Smirnov test. Based on the distribution, data are presented as mean ± standard deviation (compared with t-test/ANOVA) or median (interquartile range) (compared with Mann–Whitney U/Kruskal-Wallis test). Categorical variables were presented as numbers (%) and compared using the chi-square test. We used multivariable logistic regression to examine the associations of the PHD and its constituent food groups with sleep health indicators, with adjustment for potential confounders including age, gender, ethnicity, BMI, socioeconomic factors (income, residence, marital status, labor status), lifestyle behaviors (smoking, alcohol use, exercise), and health conditions (hypertension and diabetes). To explore potential nonlinearity in the relationship between PHD and sleep health, a restricted cubic spline (RCS) analysis was employed.

To further assess the joint association of the PHD and exercise with sleep health, participants were categorized into four groups based on their PHDI (low/high) and exercise (yes/no) status. Using participants with low PHDI and no exercise as the reference group, multivariable-adjusted odds ratios (ORs) with 95% confidence intervals (CIs) were calculated for all exposure groups. All models were adjusted for a comprehensive set of demographic, lifestyle, and clinical confounders.

To explore potential effect modification, we examined the association between the PHD and sleep health in subgroups defined by age (65–79, 80–99, or ≥100 years), gender (men or women), BMI category (<18.5, 18.5–23.9, or ≥24 kg/m^2^), alcohol consumption (yes or no), residence type (urban, town, or rural), and smoking status (yes or no). Formal tests for multiplicative interaction were conducted between the PHDI and each of these stratification variables. A sensitivity analysis was performed with additional adjustment for depression and anxiety to mitigate potential confounding from mental health factors. To explore the differential effects of PHD on short, intermediate, and long sleep duration, we conducted sensitivity analyses using intermediate sleep duration as the reference group, examining its association with both shorter and longer sleep. To evaluate the potential impact of unmeasured confounding, we calculated the E-value (23).

All analyses were performed using SAS software (version 9.4; SAS Institute Inc.), with statistical significance defined by a two-sided alpha level of 0.05.

## Results

### Characteristics of the study participants

As detailed in Supplementary Table S2, the analytical sample comprised 9,041 participants. Among them, 4,730 (52.32%) reported good sleep quality and 3,257 (36.02%) had adequate sleep duration. A significantly higher BMI was observed in participants with good versus poor sleep quality (*p* < 0.05). Conversely, participants with adequate sleep duration were significantly younger and had higher PHDI scores than those with inadequate duration (all *p* < 0.05). Similarly, those in the highest PHDI tertile were also younger but had a higher BMI than those in the lowest tertile (all *p* < 0.05) (Table 1).

**Table 1:** Baseline characteristics of participants according to tertiles of the planetary health diet index.

Variables	Planetary health diet index			*p* valve
	Tertile 1	Tertile 2	Tertile 3	
Number of participants, *n*	2,072	3,532	3,437	
Age (years)	91.00 (82.00, 100.00)	85.00 (76.00, 94.00)	80.00 (72.00, 90.00)	<0.05
BMI (kg/m^2^)	21.11 (18.77, 24.06)	22.14 (19.53, 24.80)	22.67 (20.07, 25.39)	<0.05
Gender, *n* (%)				<0.05
Men	825 (39.82)	1,552 (43.94)	1,583 (46.06)	
Women	1,247 (60.18)	1,980 (56.06)	1,854 (53.94)	
Marital status, *n* (%)				<0.05
Live with spouse	592 (28.57)	1,415 (40.06)	1,721 (50.07)	
Live without spouse	1,480 (71.43)	2,117 (59.94)	1,716 (49.93)	
Race, *n* (%)				<0.05
Han	1,999 (96.48)	3,307 (93.63)	3,221 (93.72)	
Other	73 (3.52)	225 (6.37)	216 (6.28)	
Residence, *n* (%)				<0.05
City	489 (23.60)	889 (25.17)	888 (25.84)	
Town	760 (36.68)	1,189 (33.66)	1,083 (31.51)	
Rural	823 (39.72)	1,454 (41.17)	1,466 (42.65)	
Household annual income, *n* (%)				<0.05
<30,000 yuan	978 (47.20)	1,572 (44.51)	1,428 (41.55)	
≥30,000 yuan	1,094 (52.80)	1,960 (55.49)	2,009 (58.45)	
Smoking status, *n* (%)				0.21
No	1,745 (84.22)	3,031 (85.82)	2,910 (84.67)	
Yes	327 (15.78)	501 (14.18)	527 (15.33)	
Drinking status, *n* (%)				<0.05
No	1,787 (86.25)	3,043 (86.16)	2,898 (84.32)	
Yes	285 (13.75)	489 (13.84)	539 (15.68)	
Exercise status, *n* (%)				<0.05
No	1,502 (72.49)	2,453 (69.45)	2,135 (62.12)	
Yes	570 (27.51)	1,079 (30.55)	1,302 (37.88)	
Labor status, *n* (%)				<0.05
No	525 (25.34)	885 (25.06)	949 (27.61)	
Yes	1,547 (74.66)	2,647 (74.94)	2,488 (72.39)	
Hypertension, *n* (%)				<0.05
No	843 (40.69)	1,317 (37.29)	1,172 (34.10)	
Yes	1,229 (59.31)	2,215 (62.71)	2,265 (65.90)	
Diabetes, *n* (%)				<0.05
No	1,909 (92.13)	3,150 (89.18)	2,969 (86.38)	
Yes	163 (7.87)	382 (10.82)	468 (13.62)	

BMI, body mass index.

Values are numbers (percentages) for categorical variables and median (interquartile range) for continuous variables.

### Association between PHD and sleep health

As summarized in Table 2, higher PHD was associated with better sleep health. Participants with a higher PHDI had significantly greater odds of good sleep quality (OR = 1.35, 95% CI = 1.20–1.52) and adequate sleep duration (OR = 1.36, 95% CI = 1.20–1.54) compared to those with a lower PHDI. Furthermore, each one-point increase in the PHDI was correlated with higher odds of good sleep quality and adequate sleep duration. The RCS analysis revealed a nonlinear relationship between PHDI and sleep quality, whereas no such relationship was observed with sleep duration (Supplementary Figure S1).

**Table 2:** Association of planetary health diet with sleep quality and sleep duration.

Variables	N _event_/N _total_	Model 1	Model 2	Model 3
		OR (95% CI)	OR (95% CI)	OR (95% CI)
Sleep quality				
PHDI				
T1	1,036/2,072	1.00 (Reference)	1.00 (Reference)	1.00 (Reference)
T2	1,756/3,532	0.99 (0.89, 1.10)	1.00 (0.89, 1.12)	1.02 (0.91, 1.14)
T3	1,938/3,437	1.29 (1.16, 1.44)	1.34 (1.19, 1.50)	1.35 (1.20, 1.52)
*p* for trend *		<0.05	<0.05	<0.05
Continuous **	4,730/9,041	1.04 (1.02, 1.05)	1.04 (1.03, 1.06)	1.05 (1.03, 1.06)
Sleep duration				
PHDI				
T1	628/2,072	1.00 (Reference)	1.00 (Reference)	1.00 (Reference)
T2	1,223/3,532	1.22 (1.08, 1.37)	1.13 (1.00, 1.28)	1.13 (0.99, 1.27)
T3	1,406/3,437	1.59 (1.42, 1.79)	1.39 (1.23, 1.57)	1.36 (1.20, 1.54)
*p* for trend *		<0.05	<0.05	<0.05
Continuous **	3,257/9,041	1.06 (1.05, 1.08)	1.05 (1.03, 1.06)	1.04 (1.03, 1.06)

CI, confidence interval; OR, odds ratio; PHDI, planetary health diet index; T, tertile.

Model 1: Crude model.

Model 2: Adjusted for age (years), gender (men, women), and BMI (kg/m^2^).

Model 3: Further adjusted for annual income level (≥30,000, <30,000 yuan), ethnicity (Han or others), exercise status (yes, no), labor status (yes, no), marital status (live with spouse, live without spouse), residence (city, town, or rural), smoking status (yes, no), drinking status (yes, no), hypertension (yes, no), and diabetes (yes, no).

* *p* value for linear trend calculated from category median values.

** Continuous intakes were calculated by per one unit increase.

### Associations between the food groups of the PHD and sleep health

As detailed in Supplementary Table S3, a higher intake of vegetables, fruits, soybeans, and nuts was associated with better sleep quality and adequate sleep duration, compared to rare or no consumption. Conversely, rare consumption of eggs, dairy, and sugar was linked to poorer sleep quality relative to daily intake. Additionally, a higher grain intake (≥232 g/day) was correlated with better sleep quality and adequate sleep duration compared to a low intake (<58 g/day). Interestingly, for fish, even monthly or occasional consumption (versus rare or never) was associated with adequate sleep duration.

### Combined associations of PHD and exercise with sleep health

**Figure 2:**
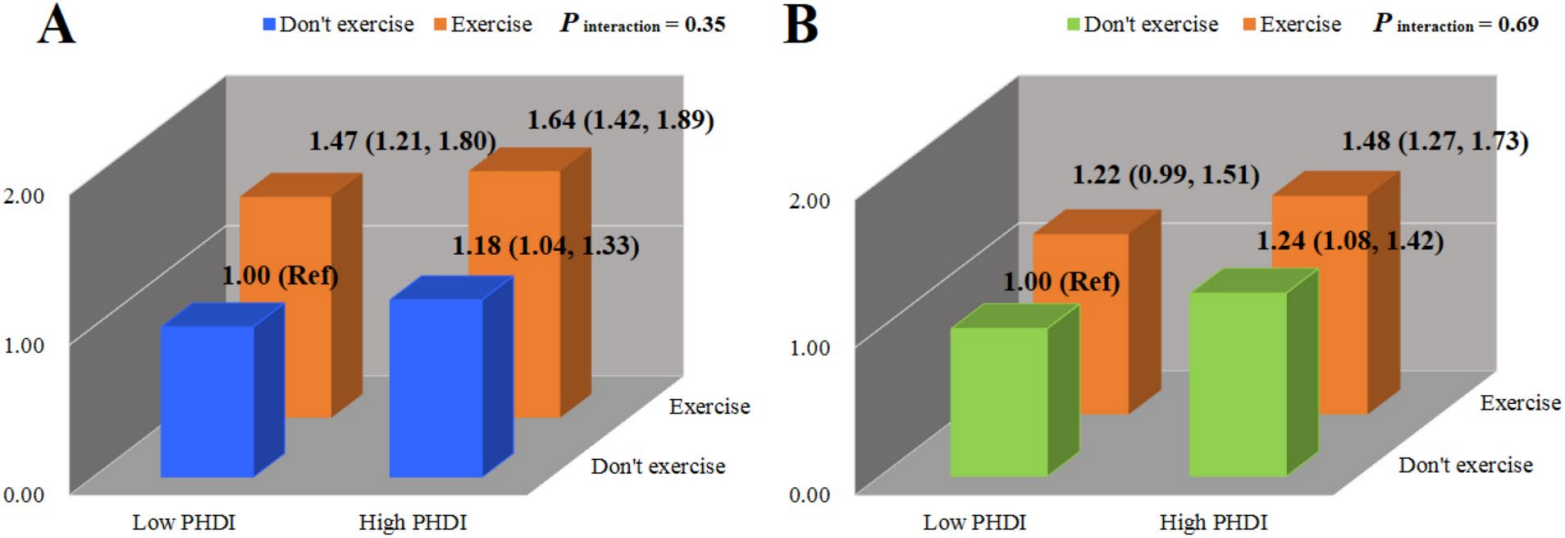
Combined effects of the planetary health diet and exercise on sleep quality **(A)** and sleep duration **(B)**. PHDI, planetary health diet index; Ref, reference. ORs and 95% CIs were calculated using multivariable logistic regression adjusted for age (years), gender (men, women), BMI (kg/m^2^), annual income level (≥30,000, <30,000 yuan), marital status (live with spouse, live without spouse), residence (city, town, or rural), ethnicity (Han or others), exercise status (yes, no), labor status (yes, no), smoke status (yes, no), drinking status (yes, no), hypertension (yes, no), and diabetes (yes, no).

Figure 2 illustrates the joint associations of PHD and exercise with sleep health. Compared to the reference group (low PHDI and no exercise), participants with high PHDI who exercised had significantly higher odds of good sleep quality (OR = 1.64, 95% CI = 1.42–1.89) and adequate sleep duration (OR = 1.48, 95% CI = 1.27–1.73). Furthermore, no significant multiplicative interaction was observed between PHDI and exercise for either sleep outcome.

### Subgroup and sensitivity analysis

Subgroup analysis revealed that the positive association between PHDI and sleep quality was observed broadly across subgroups, including those aged 65–100, urban and rural residents, individuals with obesity, and non-smokers. Conversely, the association with adequate sleep duration was more specific, being significant only in the 65–100 age group and urban/rural residents, where it was particularly pronounced (Supplementary Table S4). Notably, a significant multiplicative interaction emerged between the PHDI and both age and BMI in relation to sleep quality (Supplementary Table S4). The sensitivity analysis, which was adjusted for mental factors such as depression and anxiety, indicated that the direction of these associations was consistent with the main analysis (Supplementary Table S5). In addition, sensitivity analysis revealed that higher PHD was associated with both shorter and longer sleep duration compared to the optimal sleep duration (Supplementary Table S6). The E-value analysis suggested that the association between PHDI and sleep health could only be explained by considerable unmeasured confounding (Supplementary Table S7).

## Discussion

This community-based study indicates that higher adherence to the PHD was associated with better sleep quality and appropriate sleep duration among older adults in China. Furthermore, participants who adhered to the high PHDI and engaged in physical exercise exhibited better sleep quality and more appropriate sleep duration compared to those with the low PHDI adherence and no exercise. For clinicians and public health initiatives aimed at improving sleep in the aging population, our findings suggest that promoting a PHD, particularly in conjunction with physical exercise, may be an effective non-pharmacological approach.

This study provides novel evidence on the relationship between the PHD and sleep health. It thereby bridges a critical knowledge gap, moving beyond the well-documented benefits of PHD for conditions like cancer (24), metabolic disease (25), and cardiovascular outcomes (6, 8) to investigate its potential role in sleep quality and duration. Earlier studies have suggested that dietary patterns rich in plant-based foods were associated with better sleep quality and duration (21, 26), which was consistent with our findings. Similarly, a cross-sectional study indicated that adherence to the Mediterranean diet, characterized by high consumption of vegetables and fruits, was related to better sleep quality (27). In addition, greater adherence to the DASH diet, which is rich in fruits, soy, and nuts was linked to lower odds of insufficient sleep among Iranian employees (28). Our results broaden the current knowledge by linking a higher PHDI score, a marker of a plant-based and sustainable diet, to better sleep health among older adults. This translation of a dietary pattern into a modifiable risk factor for sleep health offers an actionable target for public health initiatives, paving the way for targeted preventive strategies to improve sleep in the aging population.

Interestingly, the positive association between the PHD and sleep quality was observed exclusively in older adults aged 65–99 years, with no statistically significant link detected among centenarians. The occurrence of this phenomenon may be attributed to inherent differences in physiological homeostasis and adaptive capacity across different age groups. Specifically, in adults aged 65–99, age-related declines in metabolic, digestive, and neuroregulatory capacity, compounded by a higher burden of comorbid conditions, may collectively increase the vulnerability of sleep architecture to physiological dysfunction (13, 29, 30). The PHD, characterized by high fiber content, minimally processed whole foods, and a balanced macronutrient-micronutrient profile, which has been associated with mitigating age-related functional decline (31). In addition, it is also plausible that the beneficial impact of these foods on sleep is mediated indirectly via their role in shaping the gut microbiota and facilitating gut-brain axis interactions (32, 33). In contrast, centenarians often possess protective genetic variants related to metabolic regulation and sleep–wake cycles (34, 35). Their lifelong dietary and lifestyle habits have fostered a highly stable microbial and hormonal environment, resulting in a resilient form of physiological homeostasis that was less susceptible to dietary influences (36). Furthermore, centenarians typically exhibit naturally fragmented and shorter sleep patterns, and their nutritional requirements and thresholds may differ from those of younger elderly individuals (37). Additionally, subgroup analyses may have been limited by sample size, reducing statistical power to detect an association in the centenarian group. Future high-quality studies focused on this exceptional population are warranted to validate our findings.

Furthermore, our study revealed a potential nonlinear association between the PHD and sleep quality. Plausible explanations for this phenomenon are as follows. In the elderly cohort, individual differences may modulate the relationship between PHD adherence and sleep quality. Older adults exhibit weaker digestive and metabolic functions compared with young-old adults (38, 39). Thus, the same level of PHD adherence may exert a more pronounced beneficial effect on sleep quality in young-old adults, whereas it shows a weaker or even opposite effect in the oldest-old population. The results of our subgroup analysis further support this finding, indicating that the association between PHD and sleep quality exists in the 65–99 age group but not in centenarians. Additionally, the methodological approach to measuring sleep quality may also contribute to this observation. Self-reported sleep quality is a subjective outcome, and its relationship with objective nutritional intake may exhibit nonlinearity due to ceiling effects (e.g., most participants rate their sleep quality as “good” or “fair”) or response bias. Future well-designed high-quality studies are warranted to validate our findings.

Joint effect analysis showed a significantly stronger association of high PHD adherence combined with physical exercise with better sleep quality and appropriate sleep duration, compared to the reference group with low PHD adherence and no physical exercise. Possible explanations for this observation may include the following. First, physical exercise reduces sympathetic nervous system dominance, promotes a relaxed state of the body, and lowers inflammation levels and oxidative stress (40, 41). Meanwhile, it is plausible that the abundance of dietary fiber and phytochemicals in the PHD underlies the observed reduction in systemic inflammation (42). It is posited that the aforementioned anti-inflammatory effects underlie the alleviation of chronic low-grade inflammation mediated by the gut-brain axis. This is significant given that chronic low-grade inflammation is strongly implicated in disrupting sleep architecture among the elderly (43, 44). Additionally, the adequate and high-quality nutrients provided by the PHD, such as antioxidant vitamins and minerals, may offer effective material support for the cellular repair and stimulation of anabolic hormone synthesis induced by exercise (45, 46). Therefore, consistently consuming the PHD and maintaining regular exercise may jointly regulate the circadian rhythm, nervous system, and inflammatory system, exerting a certain effect in counteracting the age-related multidimensional decline in sleep regulatory function.

The biological mechanisms linking PHD to sleep health were not yet fully understood, but they can be explained through specific types of food sources. The PHD were rich in high-quality foods such as fruits, vegetables, nuts, and soy, which have been associated with favorable sleep health outcomes (47, 48). Our findings on the associations between specific food groups and sleep health were consistent with the prior evidence described above. Key components of this dietary pattern, such as vegetables, whole grains, legumes, and nuts, were rich in magnesium, tryptophan, and vitamin B6, nutrients closely linked to the synthesis and rhythmic regulation of sleep-related neurotransmitters (49–51). Magnesium modulates the activity of γ-aminobutyric acid receptors, suppressing overexcitation in the central nervous system and reducing nighttime awakenings, thereby improving sleep continuity (52, 53). Tryptophan, a precursor to serotonin and melatonin, crosses the blood–brain barrier to enhance serotonin conversion in the brain, subsequently promoting melatonin secretion (54, 55). This process shortens sleep onset latency and extends the duration of deep sleep. Studies indicate that vitamin B6 accelerates the conversion of tryptophan to melatonin, further supporting stable sleep health, particularly in older adults (56). Moreover, the high dietary fiber intake derived from whole grains, vegetables, and fruits in the PHD modulates the gut microbiota to promote the production of short-chain fatty acids (SCFAs) (57). These SCFAs, in turn, inhibit the release of central inflammatory cytokines such as TNF-α and IL-6 via the gut-brain axis (58, 59). Research suggests that lower systemic inflammation reduces interference with the suprachiasmatic nucleus (the sleep center) in the hypothalamus (60). These findings warrant further mechanistic studies for validation.

To our knowledge, this study was the first to investigate the relationship between the PHD and sleep health, as well as their joint association with physical exercise. The notable strengths of this study include its nationally representative, community-based design and the implementation of comprehensive subgroup and sensitivity analyses, which collectively bolster confidence in the robustness of the findings.

This study has several limitations. First, its cross-sectional design precludes the establishment of causal inferences. Second, our exposure assessment was based on a simplified FFQ. The construction of the PHDI using broad categorical frequency scores (ranging from 0 to 3 per food group) may introduce bias relative to true variations in actual food intake. Furthermore, since exposure was self-reported via questionnaire, this approach may lead to misclassification. Further studies are needed to validate the PHDI and to confirm the observed associations. Third, the measurement of sleep outcomes relied entirely on self-report, using a single sleep quality item and relatively crude categorization of sleep duration. This may lead to loss of information and could affect statistical power and clinical interpretability. Future studies should employ standardized questionnaires (e.g., pittsburgh sleep quality index) or objective methods (e.g., polysomnography, actigraphy) to enable a more comprehensive and nuanced assessment of sleep characteristics. Fourth, as the study population consisted primarily of older Chinese adults, the findings may not be generalizable to other populations. Future longitudinal studies involving diverse countries and populations are warranted. Fifth, in our study, physical activity was ascertained by a single yes/no question, which precluded our ability to assess dose–response gradients or identify potential thresholds. In the future, it is recommended to utilize the international standardized physical activity questionnaire for assessment to ensure the identification of optimal thresholds. Finally, despite extensive adjustment in our study, residual and unmeasured confounding remain a possibility. However, the E-value analysis suggests that such confounding would need to be considerable to fully account for the observed association.

## Conclusion

Higher PHD was associated with better sleep quality and appropriate sleep duration among Chinese older adults. Participants with both high PHDI and physical exercise had the highest odds of better sleep quality and appropriate sleep duration. These findings point to the value of promoting PHD and emphasizing physical exercise to enhance sleep health in aging populations. However, these observational results must be complemented by further clinical trials or prospective studies to establish causality.

## Data Availability

The original contributions presented in the study are included in the article/Supplementary material, further inquiries can be directed to the corresponding authors.
