# Supplementary material for: Association between the planetary health diet and sleep health in older adults: findings from a national community-based study
Source: Front Nutr. 2026 Feb 4;13:1758298. doi: 10.3389/fnut.2026.1758298 (PMC12913177; doi:10.3389/fnut.2026.1758298)
Supplement: Supplementary file 1 [file Table_1.DOCX]

**Supplementary Table S1**. Calculation of planetary-health diet index.

| **Components** | | **3 points** | **2 points** | **1 point** | **0 points** |
| --- | --- | --- | --- | --- | --- |
| Emphasized intake | Vegetable | 1 | 2 | 3 | 4 |
|  | Fruit | 1 | 2 | 3 | 4 |
|  | Legumes | 1 | 2 | 3/4 | 5 |
|  | Nuts | 1 | 2 | 3/4 | 5 |
|  | Grain | >232 g | 116-232 g | 58-116 g | <58 g |
|  | Fish | 1 | 2 | 3/4 | 5 |
| Limited intake | Eggs | 5 | 3/4 | 2 | 1 |
|  | Milk and dairy | 5 | 3/4 | 2 | 1 |
|  | Sugar | 5 | 3/4 | 2 | 1 |
|  | Meat | 5 | 3/4 | 2 | 1 |
| **Total score** | 30 points | | | | |

In the categories of vegetables and fruits, the terms 1, 2, 3, and 4 denote "almost every day," "quite often," "occasionally," and "rarely or never," respectively.

In other food groups, the terms 1, 2, 3, 4, and 5 correspond to "almost every day," "not every day but at least once a week," "not every week but at least once a month," "not every month but occasionally," and "rarely or never".

Grains are scored according to the daily intake.

**Supplementary Table S2.** Baseline characteristics according to sleep quality and sleep duration.

| Characteristics | Total | Sleep quality | | *P* valve | Sleep duration | | *P* valve |
| --- | --- | --- | --- | --- | --- | --- | --- |
|  |  | Poor | Good |  | Inadequate | Adequate |  |
| Number of participants, n | 9,041 | 4,311 | 4,730 |  | 5,784 | 3,257 |  |
| Age (years) | 84.00 (75.00, 94.00) | 84.00 (75.00, 94.00) | 85.00 (75.00, 95.00) | 0.30 | 86.00 (76.00, 95.00) | 82.00 (73.00, 92.00) | < 0.05 |
| BMI (kg/m^2^) | 22.21 (19.53, 24.89) | 21.99 (19.29, 24.68) | 22.31 (19.71, 25.07) | < 0.05 | 21.97 (19.29, 24.67) | 22.50 (19.98, 25.20) | < 0.05 |
| PHDI (scores) | 16.00 (14.00, 17.00) | 16.00 (14.00, 17.00) | 16.00 (14.00, 18.00) | < 0.05 | 15.00 (14.00, 17.00) | 16.00 (14.00, 18.00) | < 0.05 |
| Gender, n (%) |  |  |  | < 0.05 |  |  | < 0.05 |
| Men | 3,960 (43.80) | 1,665 (38.62) | 2,295 (48.52) |  | 2,419 (41.82) | 1,541 (47.31) |  |
| Women | 5,081 (56.20) | 2,646 (61.38) | 2,435 (51.48) |  | 3,365 (58.18) | 1,716 (52.69) |  |
| Marital status, n (%) |  |  |  | 0.14 |  |  | < 0.05 |
| Live with spouse | 3,728 (41.23) | 1,743 (40.43) | 1,985 (41.97) |  | 2,208 (38.17) | 1,520 (46.67) |  |
| Live without spouse | 5,313 (58.77) | 2,568 (59.57) | 2,745 (58.03) |  | 3,576 (61.83) | 1,737 (53.33) |  |
| Race, n (%) |  |  |  | < 0.05 |  |  | 0.16 |
| Han | 8,527 (94.31) | 4,040 (93.71) | 4,487 (94.86) |  | 5,470 (94.57) | 3,057 (93.86) |  |
| Other | 514 (5.69) | 271 (6.29) | 243 (5.14) |  | 314 (5.43) | 200 (6.14) |  |
| Residence, n (%) |  |  |  | < 0.05 |  |  | < 0.05 |
| City | 2,266 (25.06) | 1,039 (24.10) | 1,227 (25.94) |  | 1,352 (23.37) | 914 (28.06) |  |
| Town | 3,032 (33.54) | 1,480 (34.33) | 1,552 (32.81) |  | 1,981 (34.25) | 1,051 (32.27) |  |
| Rural | 3,743 (41.40) | 1,792 (41.57) | 1,951 (41.25) |  | 2,451 (42.38) | 1,292 (39.67) |  |
| Household annual income, n (%) | |  |  | < 0.05 |  |  | < 0.05 |
| < 30,000 yuan | 3,978 (44.00) | 2,005 (46.51) | 1,973 (41.71) |  | 2,660 (45.99) | 1,318 (40.47) |  |
| ≥ 30,000 yuan | 5,063 (56.00) | 2,306 (53.49) | 2,757 (58.29) |  | 3,124 (54.01) | 1,939 (59.53) |  |
| Smoking status, n (%) |  |  |  | < 0.05 |  |  | < 0.05 |
| Non-smoker | 7,686 (85.01) | 3,762 (87.27) | 3,924 (82.96) |  | 4,957 (85.70) | 2,729 (83.79) |  |
| Current smoker | 1,355 (14.99) | 549 (12.73) | 806 (17.04) |  | 827 (14.30) | 528 (16.21) |  |
| Drinking status, n (%) |  |  |  | < 0.05 |  |  | < 0.05 |
| Non-drinker | 7,728 (85.48) | 3,796 (88.05) | 3,932 (83.13) |  | 4,980 (86.10) | 2,748 (84.37) |  |
| Current drinker | 1,313 (14.52) | 515 (11.95) | 798 (16.87) |  | 804 (13.90) | 509 (15.63) |  |
| Exercise status, n (%) |  |  |  | < 0.05 |  |  | < 0.05 |
| No | 6,090 (67.36) | 3,076 (71.35) | 3,014 (63.72) |  | 4,051 (70.04) | 2,039 (62.60) |  |
| Yes | 2,951 (32.64) | 1,235 (28.65) | 1,716 (36.28) |  | 1,733 (29.96) | 1,218 (37.40) |  |
| Labor status, n (%) |  |  |  | 0.71 |  |  | < 0.05 |
| No | 2,359 (26.09) | 1,117 (25.91) | 1,242 (26.26) |  | 1,454 (25.14) | 905 (27.79) |  |
| Yes | 6,682 (73.91) | 3,194 (74.09) | 3,488 (73.74) |  | 4,330 (74.86) | 2,352 (72.21) |  |

BMI, body mass index; PHDI, planetary health diet index.

Values are numbers (percentages) for categorical variables and median (interquartile range) for continuous variables.

**Supplementary Table S3**. Association between individual components of the planetary health diet and sleep quality and sleep duration.

| **Characteristic** | ****N _total_ (%)**** | ****Sleep quality**** | | **Sleep duration** | |
| --- | --- | --- | --- | --- | --- |
|  |  | **OR (95 % CI)** | *P* _value_ | **OR (95 % CI)** | *P* _value_ |
| **Vegetable** |  |  |  |  |  |
| Rarely or never | 305 (3.37) | 1.0 (Reference) |  | 1.0 (Reference) |  |
| Occasionally | 688 (7.61) | 0.96 (0.72, 1.29) | 0.81 | 1.26 (0.91, 1.77) | 0.16 |
| Quite often | 2,062 (22.81) | 1.11 (0.85, 1.44) | 0.45 | 1.49 (1.11, 2.02) | < 0.05 |
| Almost every day | 5,986 (66.21) | 1.44 (1.12, 1.85) | < 0.05 | 1.53 (1.16, 2.06) | < 0.05 |
| **Fruit** |  |  |  |  |  |
| Rarely or never | 2,174 (24.05) | 1.00 (Reference) |  | 1.00 (Reference) |  |
| Occasionally | 2,642 (29.22) | 1.06 (0.94, 1.19) | 0.35 | 1.16 (1.03, 1.32) | < 0.05 |
| Quite often | 2,116 (23.40) | 1.49 (1.31, 1.69) | < 0.05 | 1.34 (1.18, 1.54) | < 0.05 |
| Almost every day | 2,109 (23.33) | 1.74 (1.52, 1.99) | < 0.05 | 1.50 (1.31, 1.72) | < 0.05 |
| **Legumes** |  |  |  |  |  |
| Rarely or never | 1,277 (14.12) | 1.00 (Reference) |  | 1.00 (Reference) |  |
| Not every day but at least once a week | 2,944 (32.56) | 1.10 (0.96, 1.27) | 0.16 | 1.24 (1.07, 1.44) | < 0.05 |
| Not every week but at least once a month/  Not every month but occasionally | 3,666 (40.55) | 1.29 (1.12, 1.47) | < 0.05 | 1.32 (1.15, 1.53) | < 0.05 |
| Almost every day | 1,154 (12.76) | 1.54 (1.30, 1.82) | < 0.05 | 1.30 (1.09, 1.55) | < 0.05 |
| **Nuts** |  |  |  |  |  |
| Rarely or never | 4,754 (52.58) | 1.00 (Reference) |  | 1.00 (Reference) |  |
| Not every day but at least once a week | 2,528 (27.96) | 1.10 (1.00, 1.23) | < 0.05 | 1.20 (1.07, 1.33) | < 0.05 |
| Not every week but at least once a month/  Not every month but occasionally | 1,161 (12.84) | 1.23 (1.07, 1.41) | < 0.05 | 1.17 (1.02, 1.35) | < 0.05 |
| Almost every day | 598 (6.61) | 1.52 (1.26, 1.84) | < 0.05 | 1.29 (1.07, 1.55) | < 0.05 |
| **Grain** |  |  |  |  |  |
| <58 g | 219 (2.42) | 1.00 (Reference) |  | 1.00 (Reference) |  |
| 58-116 g | 1,058 (11.70) | 1.27 (0.93 1.74) | 0.14 | 1.19 (0.84, 1.71) | 0.33 |
| 116-232 g | 3,191 (35.29) | 1.36 (1.01, 1.84) | < 0.05 | 1.40 (1.01, 1.97) | 0.05 |
| >232 g | 4,573 (50.58) | 1.87 (1.39, 2.53) | < 0.05 | 1.64 (1.19, 2.32) | < 0.05 |
| **Fish** |  |  |  |  |  |
| Rarely or never | 1,808 (20.00) | 1.00 (Reference) |  | 1.00 (Reference) |  |
| Not every day but at least once a week | 2,849 (31.51) | 0.95 (0.84, 1.08) | 0.45 | 1.06 (0.93, 1.21) | 0.41 |
| Not every week but at least once a month/  Not every month but occasionally | 3,538 (39.13) | 1.12 (0.99, 1.26) | 0.08 | 1.25 (1.10, 1.42) | < 0.05 |
| Almost every day | 846 (9.36) | 1.18 (0.99, 1.41) | 0.06 | 1.03 (0.86, 1.24) | 0.73 |
| **Eggs** |  |  |  |  |  |
| Almost every day | 3,563 (39.41) | 1.00 (Reference) |  | 1.00 (Reference) |  |
| Not every week but at least once a month/  Not every month but occasionally | 3,095 (34.23) | 0.73( 0.65, 0.81) | < 0.05 | 0.93 (0.84, 1.04) | 0.20 |
| Not every day but at least once a week | 1,546 (17.10) | 0.63 (0.55, 0.72) | < 0.05 | 0.95 (0.83, 1.09) | 0.45 |
| Rarely or never | 837 (9.26) | 0.63 (0.53, 0.74) | < 0.05 | 0.88 (0.74, 1.04) | 0.14 |
| **Milk and dairy** |  |  |  |  |  |
| Almost every day | 2,304 (25.48) | 1.00 (Reference) |  | 1.00 (Reference) |  |
| Not every week but at least once a month/  Not every month but occasionally | 1,456 (16.10) | 0.89 (0.77, 1.02) | 0.10 | 0.91 (0.79, 1.05) | 0.20 |
| Not every day but at least once a week | 1,869 (20.67) | 0.85 (0.74, 0.97) | < 0.05 | 0.97 (0.84, 1.11) | 0.66 |
| Rarely or never | 3,412 (37.75) | 0.85 (0.75, 0.96) | < 0.05 | 0.84 (0.74, 0.95) | < 0.05 |
| **Sugar** |  |  |  |  |  |
| Almost every day | 1,083 (11.98) | 1.00 (Reference) |  | 1.00 (Reference) |  |
| Not every week but at least once a month/  Not every month but occasionally | 1,652 (18.27) | 0.82 (0.70, 0.96) | < 0.05 | 1.05 (0.89, 1.25) | 0.55 |
| Not every day but at least once a week | 2,690 (29.75) | 0.78 (0.68, 0.91) | < 0.05 | 1.13 (0.97, 1.32) | 0.12 |
| Rarely or never | 3,616 (40.00) | 0.77 (0.67, 0.89) | < 0.05 | 1.03 (0.88, 1.19) | 0.74 |
| **Meat** |  |  |  |  |  |
| Almost every day | 3,711 (41.05) | 1.00 (Reference) |  | 1.00 (Reference) |  |
| Not every week but at least once a month/  Not every month but occasionally | 3,338 (36.92) | 0.90 (0.81, 0.99) | < 0.05 | 0.99 (0.90, 1.10) | 0.97 |
| Not every day but at least once a week | 1,218 (13.47) | 0.84 (0.73, 0.96) | < 0.05 | 0.87 (0.75, 0.99) | < 0.05 |
| Rarely or never | 774 (8.56) | 0.89 (0.75, 1.05) | 0.15 | 0.90 (0.75, 1.07) | 0.23 |

CI, confidence interval; OR, odds ratio.

ORs and 95 % CIs were adjusted for age (years), gender (men, women), BMI (kg/m^2^), annual income level (≥30,000, <30,000 yuan), ethnicity (Han or others), exercise status (yes, no), labor status (yes, no), marital status (live with spouse, live without spouse), residence (city, town, or rural), smoking status (yes, no), drinking status (yes, no), hypertension (yes, no), and diabetes (yes, no).

**Supplementary Table S4.** Subgroup analyses of the association between planetary health diet and sleep quality and sleep duration.

|  | Sleep quality | | | Sleep duration | | |
| --- | --- | --- | --- | --- | --- | --- |
|  | **OR (95 % CI)** | *P* _value_ | *P* _interaction*_ | **OR (95 % CI)** | *P* _value_ | *P* _interaction*_ |
| Age (years) |  |  | < 0.05 |  |  | 0.15 |
| 65-79 | 1.65 (1.32, 2.07) | < 0.05 |  | 1.63 (1.30, 2.06) | < 0.05 |  |
| 80-99 | 1.30 (1.10, 1.53) | < 0.05 |  | 1.33 (1.11, 1.58) | < 0.05 |  |
| ≥ 100 | 0.99 (0.74, 1.31) | 0.92 |  | 1.09 (0.80, 1.49) | 0.57 |  |
| Gender |  |  | 0.46 |  |  | 0.41 |
| Male | 1.37 (1.14, 1.65) | < 0.05 |  | 1.46 (1.21, 1.76) | < 0.05 |  |
| Female | 1.32 (1.13, 1.54) | < 0.05 |  | 1.29 (1.09, 1.52) | < 0.05 |  |
| Residence |  |  | 0.10 |  |  | 0.47 |
| City | 1.33 (1.04, 1.70) | < 0.05 |  | 1.26 (0.99, 1.61) | 0.07 |  |
| Town | 1.15 (0.94, 1.41) | 0.17 |  | 1.27 (1.02, 1.57) | < 0.05 |  |
| Rural | 1.53 (1.27, 1.84) | < 0.05 |  | 1.48 (1.21, 1.81) | < 0.05 |  |
| BMI (kg/m^2^) |  |  | < 0.05 |  |  | 0.18 |
| <18.5 | 1.16 (0.91, 1.49) | 0.23 |  | 1.41 (1.08, 1.84) | < 0.05 |  |
| 18.5-23.9 | 1.13 (0.96, 1.34) | 0.14 |  | 1.29 (1.09, 1.53) | < 0.05 |  |
| ≥ 24 | 2.00 (1.60, 2.50) | < 0.05 |  | 1.45 (1.15, 1.82) | < 0.05 |  |
| Smoking status |  |  | 0.71 |  |  | 0.26 |
| No | 1.37 (1.21, 1.56) | < 0.05 |  | 1.32 (1.15, 1.51) | < 0.05 |  |
| Yes | 1.25 (0.92, 1.70) | 0.15 |  | 1.63 (1.19, 2.23) | < 0.05 |  |
| Drinking status |  |  | 0.24 |  |  | 0.10 |
| No | 1.33 (1.17, 1.51) | < 0.05 |  | 1.29 (1.13, 1.47) | < 0.05 |  |
| Yes | 1.49 (1.08, 2.07) | < 0.05 |  | 1.95 (1.38, 2.75) | < 0.05 |  |

BMI, body mass index; CI, confidence interval; OR, odds ratio.

ORs and 95 % CIs were adjusted for age (years), gender (men, women), BMI (kg/m^2^), annual income level (≥30,000, <30,000 yuan), ethnicity (Han or others), exercise status (yes, no), labor status (yes, no), marital status (live with spouse, live without spouse), residence (city, town, or rural), smoking status (yes, no), drinking status (yes, no), hypertension (yes, no), and diabetes (yes, no).

* indicates *P* for interaction between strata and planetary health diet index.

**Supplementary Table S5.** Sensitivity analyses: adjusted ORs and 95% CIs for the associations between the planetary health diet and sleep quality and sleep duration, after further adjustment for depression and anxiety.

| **Variables** | **N _event_/N _total_** | **Model 1** | **Model 2** | **Model 3** |
| --- | --- | --- | --- | --- |
|  |  | **OR (95 % CI)** | **OR (95 % CI)** | **OR (95 % CI)** |
| **Sleep quality** | | | | |
| **PHDI** | | | | |
| T1 | 829/1,649 | 1.00 (Reference) | 1.00 (Reference) | 1.00 (Reference) |
| T2 | 1,518/3,021 | 0.99 (0.89, 1.13) | 1.00 (0.88, 1.13) | 1.01 (0.89, 1.15) |
| T3 | 1,775/3,073 | 1.35 (1.20, 1.53) | 1.39 (1.22, 1.58) | 1.31 (1.14, 1.49) |
| *P* for trend * |  | < 0.05 | < 0.05 | < 0.05 |
| Continuous ** | 4,122/7,743 | 1.05 (1.03, 1.06) | 1.05 (1.03, 1.07) | 1.04 (1.02, 1.06) |
| **Sleep duration** | | | | |
| **PHDI** | | | | |
| T1 | 518/1,649 | 1.00 (Reference) | 1.00 (Reference) | 1.00 (Reference) |
| T2 | 1,074/3,021 | 1.20 (1.06, 1.37) | 1.13 (0.99, 1.29) | 1.12 (0.98, 1.28) |
| T3 | 1,291/3,073 | 1.58 (1.40, 1.80) | 1.39 (1.22, 1.59) | 1.31 (1.14, 1.50) |
| *P* for trend * |  | < 0.05 | < 0.05 | < 0.05 |
| Continuous ** | 2,883/7,743 | 1.06 (1.05, 1.08) | 1.05 (1.03, 1.06) | 1.04 (1.02, 1.05) |

CI, confidence interval; OR, odds ratio; PHDI, planetary health diet index; T, tertile.

Model 1: Crude model;

Model 2: Adjusted for age (years), gender (men, women), and BMI (kg/m^2^);

Model 3: Further adjusted for annual income level (≥30,000, <30,000 yuan), ethnicity (Han or others), exercise status (yes, no), labor status (yes, no), marital status (live with spouse, live without spouse), residence (city, town, or rural), smoking status (yes, no), drinking status (yes, no), hypertension (yes, no), diabetes (yes, no), depression (yes, no), and anxiety (yes, no).

* *P* value for linear trend calculated from category median values.

** Continuous intakes were calculated by per one unit increase.

**Supplementary Table S6.** Sensitivity analyses: adjusted ORs and 95% CIs for the associations between the planetary health diet and sleep duration (short sleep versus moderate sleep and long sleep versus moderate sleep).

| **Variables** | **N _event_/N _total_** | **Model 1** | **Model 2** | **Model 3** |
| --- | --- | --- | --- | --- |
|  |  | **OR (95 % CI)** | **OR (95 % CI)** | **OR (95 % CI)** |
| **Sleep duration (short sleep versus moderate sleep)** | | | | |
| **PHDI** | | | | |
| T1 | 628/1,395 | 1.00 (Reference) | 1.00 (Reference) | 1.00 (Reference) |
| T2 | 1,223/2,585 | 1.10 (0.96, 1.25) | 1.07 (0.94, 1.23) | 1.07 (0.93, 1.23) |
| T3 | 1,406/2,621 | 1.41 (1.24, 1.61) | 1.36 (1.18, 1.56) | 1.34 (1.16, 1.54) |
| *P* for trend * |  | < 0.05 | < 0.05 | < 0.05 |
| Continuous ** | 3,257/6,601 | 1.05 (1.03, 1.06) | 1.04 (1.03, 1.06) | 1.04 (1.02, 1.06) |
| **Sleep duration (long sleep versus moderate sleep)** | | | | |
| **PHDI** | | | | |
| T1 | 628/1,305 | 1.00 (Reference) | 1.00 (Reference) | 1.00 (Reference) |
| T2 | 1,223/2,170 | 1.39 (1.21, 1.60) | 1.19 (1.02, 1.37) | 1.18 (1.02, 1.37) |
| T3 | 1,406/2,222 | 1.86 (1.62, 2.13) | 1.38 (1.19, 1.60) | 1.35 (1.16, 1.57) |
| *P* for trend * |  | < 0.05 | < 0.05 | < 0.05 |
| Continuous ** | 3,257/5,697 | 1.09 (1.07, 1.11) | 1.05 (1.03, 1.07) | 1.04 (1.02, 1.06) |

CI, confidence interval; PHDI, planetary health diet index; OR, odds ratio; T, tertile.

Model 1: Crude model;

Model 2: Adjusted for age (years), gender (men, women), and BMI (kg/m^2^);

Model 3: Further adjusted for annual income level (≥30,000, <30,000 yuan), ethnicity (Han or others), exercise status (yes, no), labor status (yes, no), marital status (live with spouse, live without spouse), residence (city, town, or rural), smoking status (yes, no), drinking status (yes, no), hypertension (yes, no), and diabetes (yes, no).

* *P* value for linear trend calculated from category median values.

** Continuous intakes were calculated by per one unit increase.

**Supplementary Table S7.** Sensitivity analyses: E-values for the association between the planetary health diet and sleep quality and sleep duration.

| Outcomes | Exposure | E-values* for OR (and for CI) |
| --- | --- | --- |
| Sleep quality | PHDI | 2.04 (1.69) |
| Sleep duration | PHDI | 2.06 (1.69) |

CI, confidence interval; OR, odds ratio; PHDI, planetary health diet index.

* E-value represents the minimum strength of association needed between an unmeasured confounder and both the exposure and the outcome to fully explain away the exposure-outcome association.**
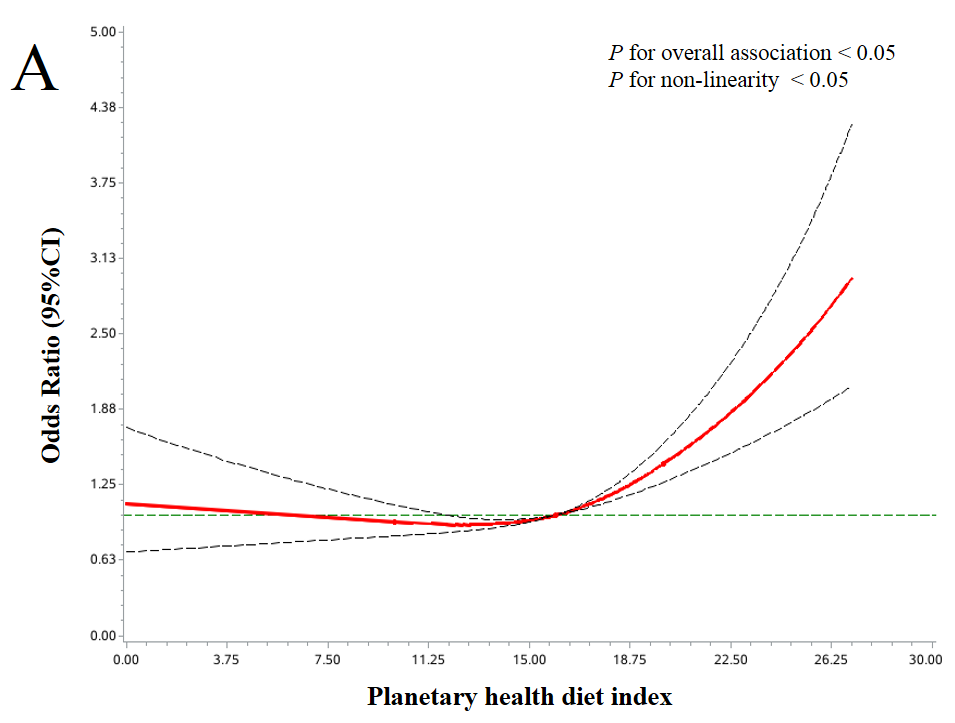
**
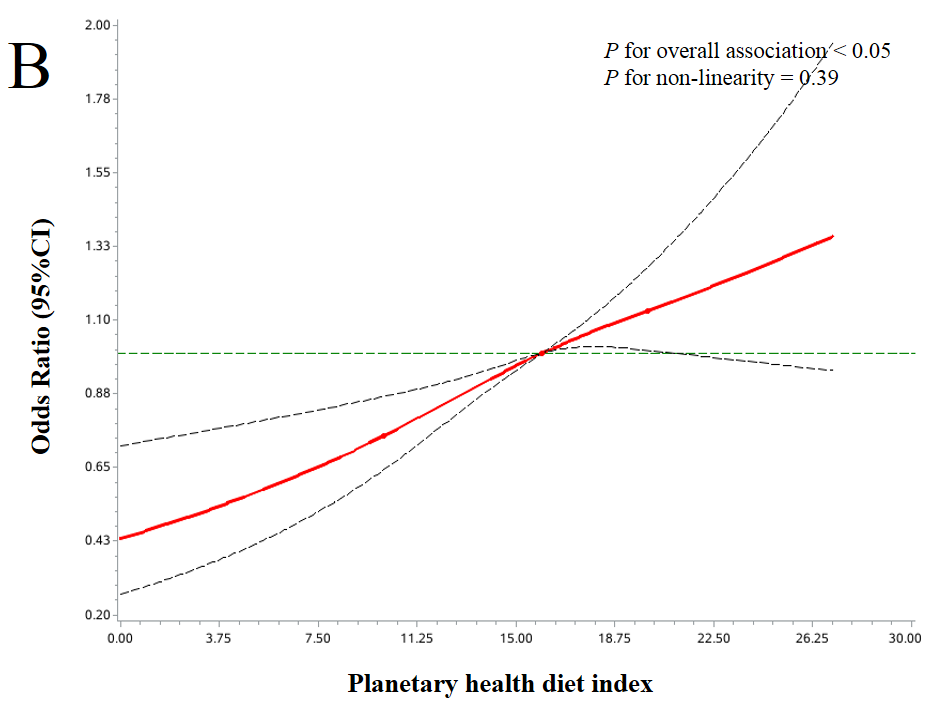


**Supplementary Figure S1.** Restricted cubic splines for planetary health diet and sleep quality (A) and sleep duration (B).

The curve was adjusted for age (years), gender (men, women), BMI (kg/m^2^), annual income level (≥30,000, <30,000 yuan), ethnicity (Han or others), exercise status (yes, no), labor status (yes, no), marital status (live with spouse, live without spouse), residence (city, town, or rural), smoking status (yes, no), drinking status (yes, no), hypertension (yes, no), and diabetes (yes, no).
